# Primary gastrointestinal stromal tumor of the liver: a case report and review of the literature

**DOI:** 10.1186/s40792-016-0218-6

**Published:** 2016-09-01

**Authors:** Takeshi Nagai, Kazumitsu Ueda, Hiroyuki Hakoda, Shinya Okata, Shoko Nakata, Tetsuro Taira, Shigeo Aoki, Hideyuki Mishima, Akihiro Sako, Tsunehiko Maruyama, Minoru Okumura

**Affiliations:** Department of Surgery, Hitachi General Hospital, 2-1-1 Jonan-cho, Hitachi, Ibaraki 317-0077 Japan

**Keywords:** Gastrointestinal stromal tumors, Extra-gastrointestinal stromal tumors, Primary hepatic tumor, Interstitial Cajal-like cells, Telocytes

## Abstract

**Background:**

Recently, gastrointestinal stromal tumors that have developed outside of the digestive tract have been reported. These tumors are collectively termed extra-gastrointestinal stromal tumors. Extra-gastrointestinal stromal tumors can also develop in the liver. Only eight case reports involving primary GIST of the liver have been published. We report a case and review the literature regarding this disease.

**Case presentation:**

A 70-year-old woman with a past history of gastric cancer visited our hospital for regular inspection. With extensive radiological imaging, a computed tomography scan revealed a mass with a size of 6.8 cm in the lateral segment of the liver. ^18^F-Fluoro-2-deoxyglucose positron emission tomography revealed no other malignancies except for the liver tumor. Because the lesion was suspected of being a primary malignant hepatic tumor, lateral segmentectomy was performed. The immunohistochemical analysis supported the diagnosis of gastrointestinal stromal tumors in the liver. The patient has had no evidence of recurrence during the 10-month follow-up period; imatinib chemotherapy was not administered.

**Conclusions:**

Primary hepatic gastrointestinal stromal tumors had no characteristics that distinguished them from ordinary tumors in imaging examinations. Primary gastrointestinal stromal tumors might have developed from interstitial Cajal-like cells.

## Background

Gastrointestinal stromal tumors (GISTs) are thought to arise from the interstitial cells of Cajal (ICCs) located in the gastrointestinal mesenchyme. The current diagnosis of GISTs is based on histological and immunohistochemical criteria, the most important of which is the expression of the receptor tyrosine kinase, KIT (CD117, c-Kit). GISTs are typically found in the gastrointestinal tract, including the stomach, small intestine, colorectum, and esophagus [1–4]. The number of case reports involving GISTs in the extra-gastrointestinal sites have been increasing. These cases are designated as extra-gastrointestinal stromal tumors (EGISTs) [5]. However, ICCs have not been identified in the liver; thus, primary GISTs occurring in the liver have been evaluated.

From around the year 2000, many groups have become interested in whether or not ICCs are present outside of the gastrointestinal tract; peculiar interstitial cells have been found in the upper and lower urinary tracts, blood vessels, pancreas, and other sites. Such cells have been named interstitial Cajal-like cells (ICLCs) [6]. Furthermore, ICLCs have been discovered in human liver fibrosis by Fu et al. [7]; therefore, primary GISTs might originate from the liver. We report a rare case of primary hepatic GIST and a review of the literature.

## Case presentation

A Japanese female patient underwent distal gastrectomy with regional lymph node dissection for gastric cancer when she was 63 years old. The cancer was a poorly differentiated adenocarcinoma with serosa invasion, and lymph node involvement was found. However, no liver tumor was detected during radiological examination at the initial operation. In addition, extensive microscopic examination of the resected stomach revealed no GISTs, with the exception of gastric adenocarcinoma.

**Fig. 1:**
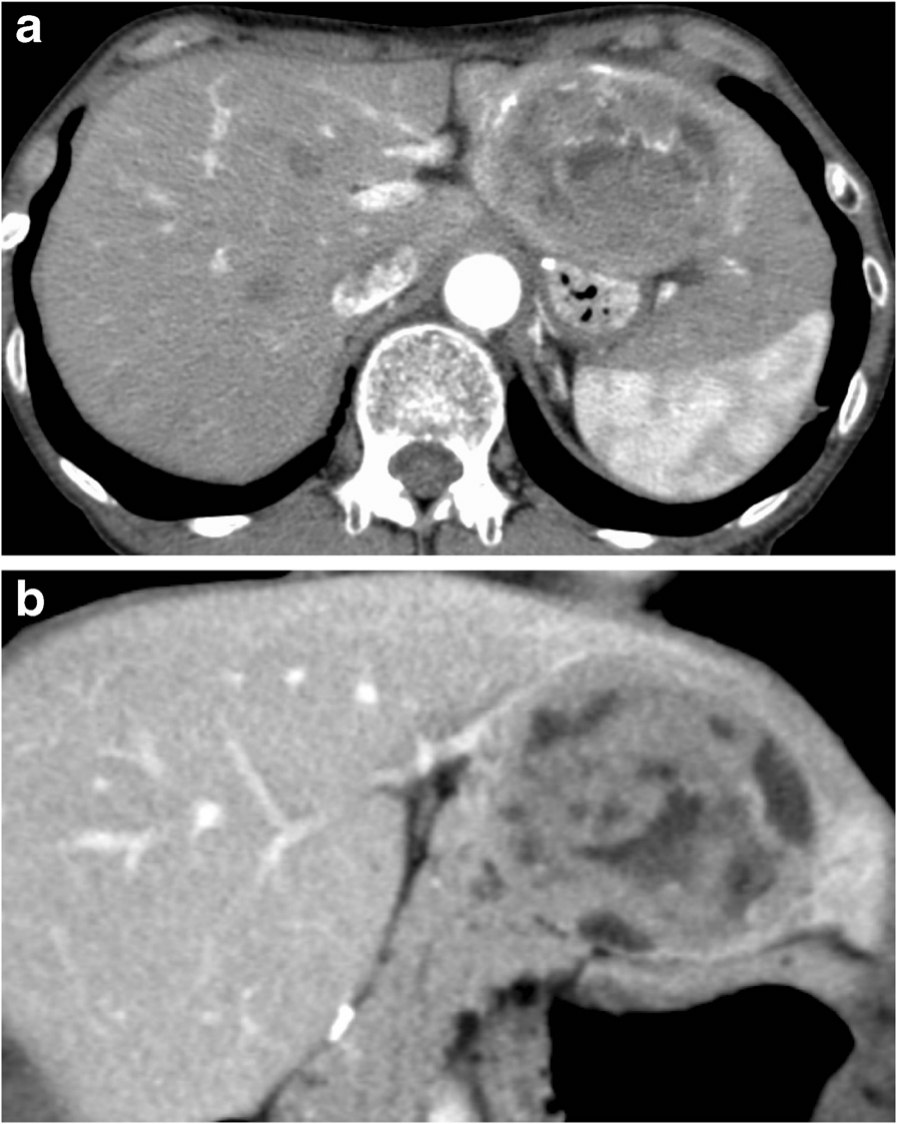
CT scan showing a large and round mass in the left hepatic lobe. The irregular low-density area resembling a mosaic pattern was found during the arterial (**a**) and portal phases (**b**)

**Fig. 2:**
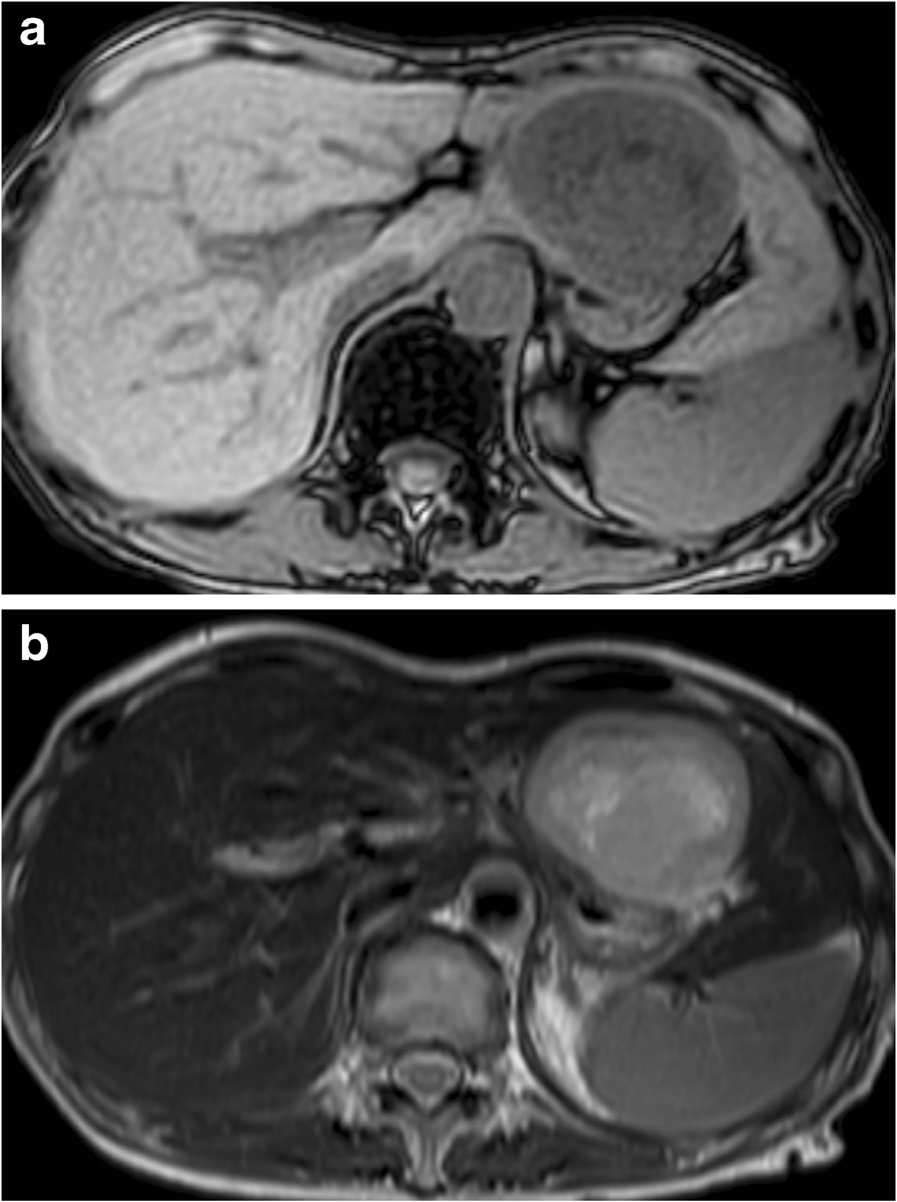
MRI scan showing a low- and high-intensity tumor on T1-weighted phase images (**a**) and T2-weighted phase images (**b**), respectively

**Fig. 3:**
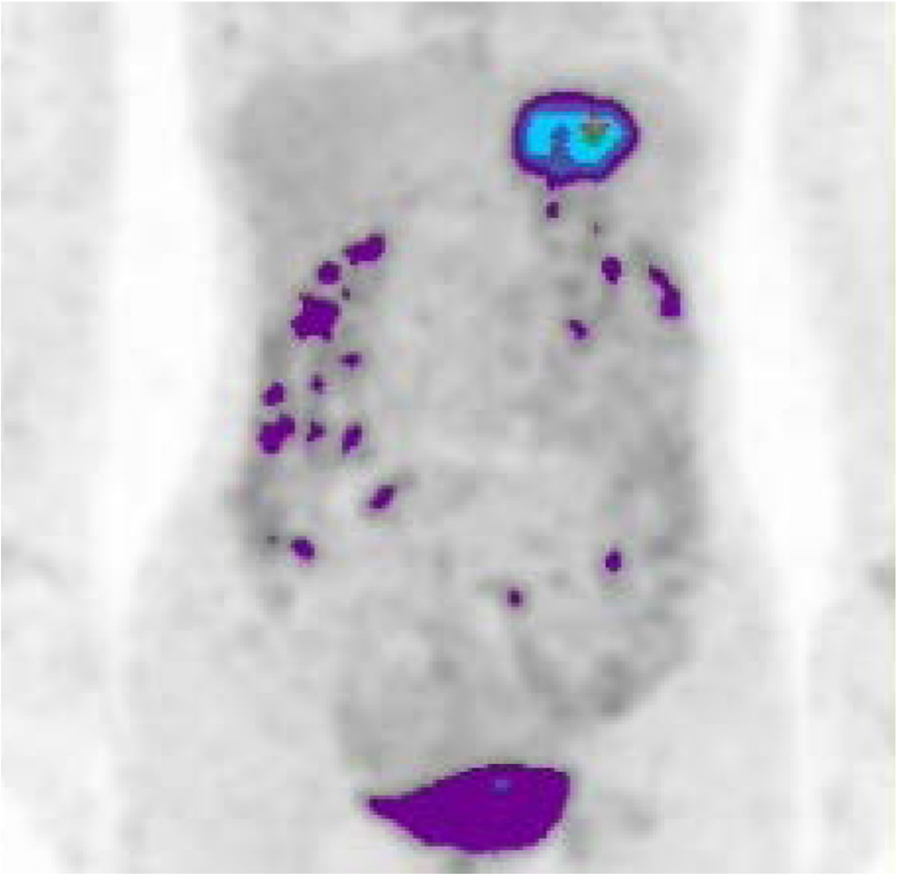
^18^F-FDG PET image showing avid FDG accumulation in the left hepatic mass (maximum standardized uptake value = 6.3)

Seven years after surgery, an abdominal computed tomography (CT) scan revealed a 6-cm mass in the left lateral segment of the liver, and peripheral enhancement in the arterial phase with a heterogenous appearance (Fig. 1a, b). The left portal vein and bile ducts in the lateral segment were not involved. Magnetic resonance imaging (MRI) showed a well-defined mass with hypointensity relative to the liver parenchyma on T1-weighted images (Fig. 2a) and moderate hyperintensity on T2-weighted images (Fig. 2b). The bile ducts in the left lateral sector were not dilated. The mass showed hyperintensity on diffusion-weighted images. In the late dynamic and hepatobiliary phases, the hepatic tumor exhibited clear hypointensity on ethoxybenzyl diethylenetriaminepentaacetic acid-MRI. ^18^F-Fluoro-2-deoxyglucose (^18^F-FDG) positron emission tomography (PET) was performed to rule out the presence of metastases throughout the body. The hepatic mass showed ^18^F-FDG uptake with a maximum standardized uptake value of 6.3; however, no other sites of hot ^18^F-FDG uptake were demonstrated (Fig. 3). Upper and lower gastrointestinal endoscopic examinations did not suggest the presence of any other neoplastic lesions. Laboratory findings including levels of serum carcinoembryonic antigen, carbohydrate antigen 19-9, and alpha-fetoprotein were within normal limits.

**Fig. 4:**
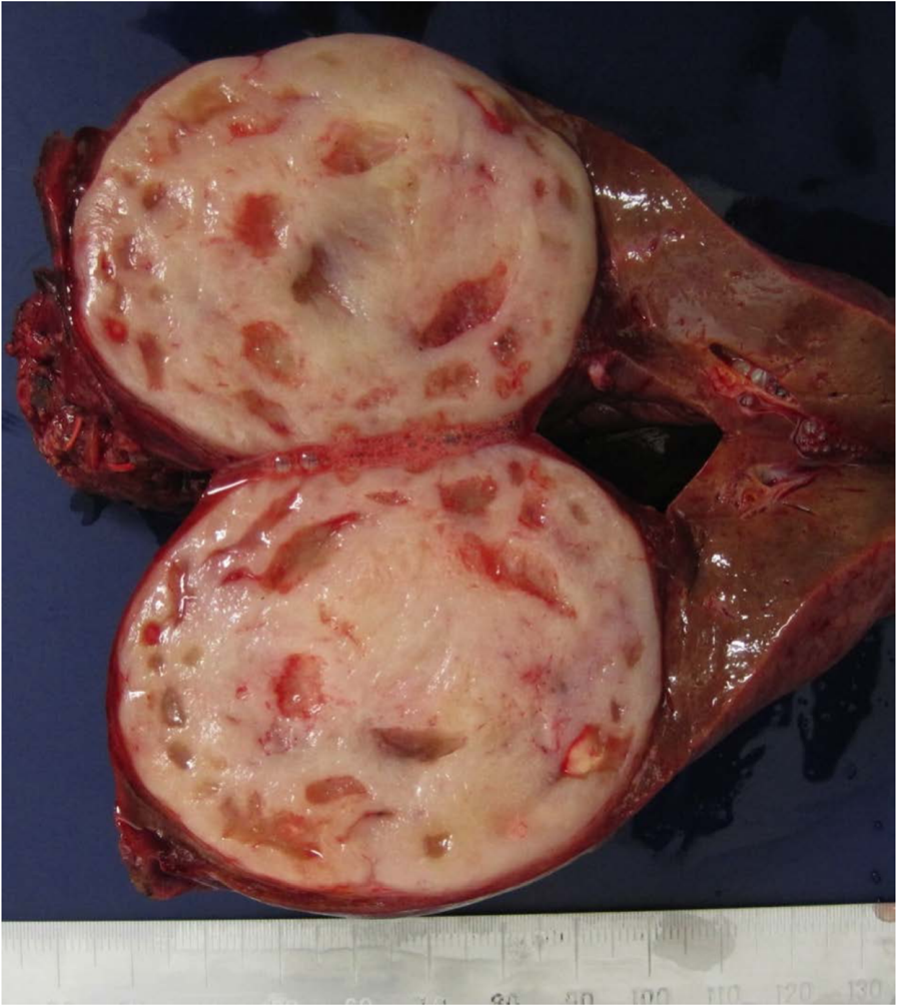
Photograph showing the gross appearance of the dissected tumor. On evaluation, the hepatic tumor was well circumscribed and measured 6.8 × 5.5 cm

The patient underwent left lateral segmentectomy of the liver because of the possibility of a primary hepatic malignancy. Intraoperative findings indicated that the hepatic tumor was palpable with a hard elastic consistency and had no lymph node involvement; no gastrointestinal tumor was found in the abdominal cavity. The hepatic tumor and the adjacent small intestine adhered firmly to each other, and thus, combined resection was performed. On gross evaluation, the hepatic tumor was well circumscribed and measured 6.8 × 5.5 cm. In cross section, the tumor had a whitish-tan color to the cut surface and focal necrotic and cystic portions (Fig. 4). No malignancies were detected in the resected adjacent small intestine.

**Fig. 5:**
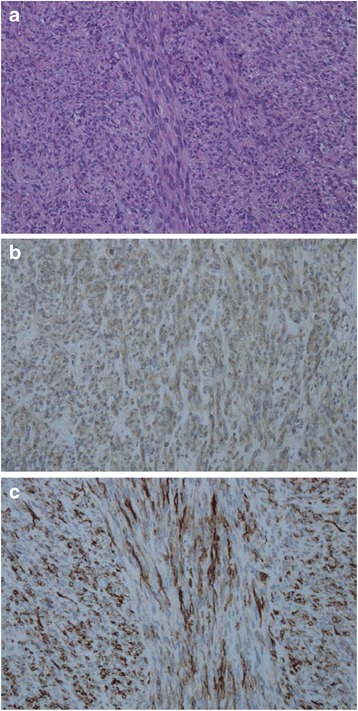
Photomicrographs of stained tumor sections. Microscopically, the tumor consisted of spindle cells with pleomorphic nuclei arranged into fascicles (hematoxylin-eosin stain, ×200) (**a**). Immunohistochemically, the tumor cells were diffusely positive for KIT (**b**) and CD34 (**c**) (×200)

Microscopically, the hepatic tumor was composed of spindle cells with pleomorphic nuclei arranged into short fascicles. The mitotic count was 35–40 mitoses per 50 high-power fields (HPFs) (Fig. 5a). Immunohistochemical staining for KIT, CD34, S-100, smooth muscle actin, and desmin was performed. Positive results were observed for KIT and CD34 (Fig. 5b, c). The pathological findings indicated the presence of GISTs in the liver. Fletcher’s risk score indicated advanced-stage disease [1].

Adjuvant therapy such as imatinib was not administered continuously because of adverse events involving general malaise and anorexia. The patient recovered well from the side effects and exhibited no evidence of recurrence during the 10-month follow-up period.

No other tumors except for the hepatic tumor were identified pre- and intra-operatively in the present case; moreover, no neoplastic lesions were identified after surgical treatment without chemotherapy. We made a final diagnosis of primary hepatic GIST.

### Discussion

GISTs are specific mesenchymal tumors located in regions of the gastrointestinal tract such as in the stomach (60–70 %), small intestine (20–25 %), colon and rectum (5 %), and esophagus (<5 %) [2]. However, in recent years, GISTs have been reported outside of the gastrointestinal tract as apparent primary tumors. They can also occur in the omentum, mesentery, retroperitoneum, urinary bladder, ureter, uterus, pancreas, and gall bladder [8–16]. They are designated as EGISTs [5]. According to Miettinen and Lasota, the frequency of EGISTs is ≤1 % of all GISTs of defined origin [17]. A PubMed search was performed using the keywords: “gastrointestinal stromal tumors,” “extra-gastrointestinal stromal tumors,” “primary hepatic tumor,” and limited to reports on human adults and English and Japanese-language publications, including case reports. Primary GIST of the liver is a type of EGIST; only eight previous case studies involving this tumor have been reported in the literature until 2015 [18–25]. The clinicopathological features and treatment outcomes of the reported primary hepatic GISTs, including the present case, are detailed in Table 1. The patients included six men and three women whose ages ranged from 17 to 79 years (mean, 56 years). There was a distinct predominance of male patients. Four of the nine patients had been asymptomatic, but five had presented with shortness of breath, abdominal fullness, loss of appetite, hypochondriac pain, nausea, and indigestion. The tumors ranged in size from 5.1 to 20 cm with a median size of 9.5 cm. Seven patients had unilobar tumors, and eight patients had solitary tumors in the liver.

**Table 1:** The clinicopathological findings and treatment outcomes of reported cases of primary hepatic gastrointestinal stromal tumors

	Author	Year	Age	Sex	Presentation	Location	Size (cm)	Cell type	Mitotic counts (no./50 HPF)	CT	MRI (T1/T2/DWI/hepatobiliary phase)	PET (SUV max)	Procedure	Recurrence (treatment)	Outcome
1	Hu et al. [18]	2003	79	F	Shortness of breath	Right lobe	15	Spindle	20	Low	NA	NA	R-HTx	Hepatic hilar LN (Surgery)	Alive (20 months)
2	De Chiara et al. [19]	2006	37	M	Asymptom	S5	18	Spindle	20	Low	NA	NA	Partial HTx	Multiple lung meta (imatinib)	Alive (36 months)
3	Ochiai et al. [20]	2009	30	M	Abdominal fullness	Bilateral lobe	>10	Mixed	75	Low	NA	NA	(1) L-Trisegmentec tomy (2) Partial HTx and gastrectomy (3) Partial HTx and resection of the thoracic operative scar	(1) Residual liver (surgery) (2) Residual liver and thoracic operative scar (surgery and imatinib)	Alive (66 months)
4	Luo et al. [21]	2009	17	M	Asymptom	Anterior segment	5.1	Spindle	0	Low	NA	NA	RFA	NED (none)	Alive (3 months)
5	Yamamoto et al. [22]	2010	70	M	Loss of appetite	Left lobe	20	Epithelioid	1	Low	NA	NA	L-HTx	NED (NA)	NA
6	Bo et al. [23]	2014	56	M	Asymptom	Right lobe	9.5	Spindle	<5	Low	High/low/−/−	NA	Central HTx	NED (none)	Alive (12 months)
7	Louis et al. [24]	2014	55	F	Hypochondriac pain	S3/S2/S6/S8	18/6/6/6	Spindle	10	Low	NA	Positive	Segmentectomy (S3) and partial resection ×3	NED (imatinib)	Alive (6 months)
8	Kim et al. [25]	2014	71	M	Nausea and indigestion	Lateral segment and peritoneal seeding	7	Spindle	30	Low	Low/high/high/low	Positive (6.9)	Lateral segmentectomy, resection of disseminated tumors	Residual peritoneal tumor (imatinib)	Dead (19 months)
9	present case	2015	70	F	Asymptom	Lateral segment	6.8	Spindle	35	Low	Low/high/high/low	Positive (6.3)	Lateral segmentectomy	NED (none)	Alive (6 months)

*HTx* hepatectomy, *RFA* radio-frequency ablation, *NA* not available, *NED* no evidence of disease

All hepatic GISTs demonstrated hypoattenuation on CT scans. MRI was performed for three patients, but no characteristic findings were obtained. ^18^F-Fluorodeoxyglucose positron emission tomography (^18^FDG-PET) was performed in three patients, and high accumulation of ^18^FDG was found in their hepatic tumors. All patients had undergone surgery, with the exception of only one who had been treated with radiofrequency ablation. Morphologically, GISTs can be subdivided into spindle cells, epithelioid cells, and mixed types. Seven of the nine cases were the spindle cell type, one was the epithelioid cell type, and one was the mixed cell type.

The mitotic count in the tumor ranged from 0 to 75 per 50 HPFs, with a median of 20 per 50 HPFs. Fletcher et al. [1] proposed a “risk of aggressive behavior” classification of GISTs based only on the tumor size and HPF mitotic count. The risk stratification divides tumors into very low-, low-, intermediate-, and high-risk categories based on size (<2, 2–5, 5–10, and >10 cm) and on the number of mitoses per 50 HPFs, typically reported as <5, 5–10, or >10. The mitotic counts were associated with the recurrence and metastasis of the disease. According to a classification that estimated the risk of aggressive behavior, five of nine cases showed high-risk tumors, including the present case. The histopathological and immunohistochemical characteristics of EGISTs are similar to those of GISTs [23]. Five cases had high mitotic counts of over 20 per 50 HPFs and, with the exception of the present case, had developed metastases or recurrence.

Four patients had received imatinib chemotherapy, one patient underwent adjuvant therapy, two of three patients with recurrence were effectively treated and remained alive for 36 and 66 months, and the other patient died of the disease. Patients with recurrent tumors could be expected to achieve long-term survival if they had received chemotherapies continuously.

The present case can be considered a primary hepatic GIST because of the following two findings. First, preoperative gastroscopy and colonoscopy imaging studies and extensive pathological examination of the resected stomach failed to detect any other GISTs, except for a hepatic tumor. Second, there was no evidence of recurrence and no neoplastic lesions after hepatectomy in the absence of adjuvant chemotherapy. ICCs are normally present in the myenteric plexus of the gastrointestinal tract, and they have not been identified in the liver. Consequently, is it possible that primary GISTs can occur in the liver? We initially suspected that primary hepatic GISTs might originate from the biliary system in the liver. Ahmadi et al. reported that ICCs were identified in the extrahepatic bile ducts [10]; unfortunately, the existence of ICCs was not recognized in intrahepatic bile ducts. However, Popescu et al. demonstrated that ICCs are present in human exocrine pancreas; such cells were named pancreatic ICC and later called ICLCs [8]. Subsequently, ICLCs were identified in many sites such as the following: the upper and lower urinary tracts, blood vessels, pancreas, male and female reproductive tracts, mammary glands, placenta, the heart, and the gut. Recently, ICLCs have been mostly referred to as telocytes (TCs) [6]. Padhi et al. reviewed 19 reported cases of pancreatic extra-gastrointestinal stromal tumors and concluded that with the discovery and characterization of pancreatic TCs, the origin of stromal tumors reminiscent of GIST seemed a real possibility [26]. Furthermore, Fu et al. have reported that TCs were identified in the liver and were diminished in liver fibrosis [7]. Regardless, TCs have not been identified in the normal liver; primary GISTs might have developed from TCs in the liver. Further investigations regarding the relationship between hepatic GISTs and TCs are necessary.

## Conclusions

Primary GIST of the liver is very rare, and only eight cases have been previously reported. Primary hepatic GISTs had no characteristics to distinguish them from ordinary ones on CT and MRI scans and also regarding histopathological findings. Primary hepatic GISTs might have developed from ICLCs, but further investigations are necessary.

## Abbreviations

CT: Computed tomography
FDG-PET: ^18^F-Fluorodeoxyglucose positron emission tomography
MRI: Magnetic resonance imaging
